# Targeting xCT, a cystine-glutamate transporter induces apoptosis and tumor regression for KSHV/HIV-associated lymphoma

**DOI:** 10.1186/1756-8722-7-30

**Published:** 2014-04-04

**Authors:** Lu Dai, Yueyu Cao, Yihan Chen, Chris Parsons, Zhiqiang Qin

**Affiliations:** 1 Research Center for Translational Medicine and Key Laboratory of Arrhythmias of the Ministry of Education of China, East Hospital, Tongji University School of Medicine, 150 Jimo Road, Shanghai 200120, China; 2 Department of Microbiology/Immunology/Parasitology, Louisiana State University Health Sciences Center, Louisiana Cancer Research Center, 1700 Tulane Ave., New Orleans, LA 70112, USA; 3 Department of Medicine, Louisiana State University Health Sciences Center, Louisiana Cancer Research Center, 1700 Tulane Ave., New Orleans, LA 70112, USA

**Keywords:** KSHV, Herpesvirus, xCT, Lymphoma

## Abstract

Kaposi’s sarcoma-associated herpesvirus (KSHV) is the etiological agent of primary effusion lymphoma (PEL), which represents a rapidly progressing malignancy arising in HIV-infected patients. Conventional chemotherapy for PEL treatment induces unwanted toxicity and is ineffective — PEL continues to portend nearly 100% mortality within a period of months, which requires novel therapeutic strategies. The amino acid transporter, xCT, is essential for the uptake of cystine required for intracellular glutathione (GSH) synthesis and for maintaining the intracellular redox balance. Inhibition of xCT induces growth arrest in a variety of cancer cells, although its role in virus-associated malignancies including PEL remains unclear. In the current study, we identify that xCT is expressed on the surface of patient-derived KSHV^+^ PEL cells, and targeting xCT induces caspase-dependent cell apoptosis. Further experiments demonstrate the underlying mechanisms including host and viral factors: reducing intracellular GSH while increasing reactive oxygen species (ROS), repressing cell-proliferation-related signaling, and inducing viral lytic genes. Using an immune-deficient xenograft model, we demonstrate that an xCT selective inhibitor, Sulfasalazine (SASP), prevents PEL tumor progression *in vivo*. Together, our data provide innovative and mechanistic insights into the role of xCT in PEL pathogenesis, and the framework for xCT-focused therapies for AIDS-related lymphoma in future.

## Introduction

The oncogenic γ-herpesvirus known as the Kaposi’s sarcoma-associated herpesvirus (KSHV) is a principal causative agent of cancer arising in patients with compromised immune systems [1]. One of these cancers, primary effusion lymphoma (PEL), comprises transformed B cells harboring KSHV episome and arises preferentially within the pleural or peritoneal cavities of patients infected with HIV [2]. PEL is a rapidly progressing malignancy with a median survival time of approximately 6 months [3]. Currently, combinational chemotherapy is the standard of care for PEL, and cyclophosphamide, doxorubicin, vincristine, and prednisone (CHOP) regimens are considered first-line therapy [4,5]. However, the myelosuppressive effects of systemic cytotoxic chemotherapy synergize with those caused by antiretroviral therapy or immune suppression [3,4,6]. Several novel approaches for PEL therapy have been reported in recent studies and increase survival for some patients, but a lack of sufficient safety and efficacy data have precluded their routine use. The proteasome inhibitor bortezomib and the combination of arsenic trioxide and interferon both suppress the NF-κB activation and may work synergistically with cytotoxic chemotherapy to reduce PEL viability [7,8]. Unfortunately, proteasome inhibition and arsenic incur significant toxicities limiting their clinical application. The mammalian target of rapamycin (mTOR) inhibitor, sirolimus inhibits PEL cell growth in a murine xenograft model [7], but which paradoxically induces expression of the serine/threonine kinase Akt and tumor cell growth, resulting in treatment failures [8]. Recently, we report that inhibition of sphingosine kinase 2 (SphK2) by a novel compound, ABC294640, effectively prevents and represses PEL tumor progression *in vivo*[9]. Even though, novel targeted, safer and more effective strategies are urgently needed for PEL treatment.

The x_c_^−^ antiporter, consisting of xCT (also named as SLC7A11) and its chaperone CD98, functions as a Na^+^-independent electroneutral exchange system for cystine/glutamate [10]. Expression of xCT on the cell membrane is essential for the uptake of cystine required for intracellular glutathione (GSH) synthesis, which plays an important role in maintaining the intracellular redox balance [11,12]. Cystine/cysteine represents an essential amino acid for many cancer cells and its uptake from the microenvironment is crucial for their growth and viability. Therefore, xCT is highly expressed by a variety of malignant tumors [13-16], and also contributes to multidrug resistance for cancer cells [17,18].

Interestingly, xCT has been also identified as a fusion-entry receptor for KSHV and mediated KSHV entry either in isolation or as part of a complex with other receptors for the virus [19,20]. Recently, our study has demonstrated that xCT is upregulated within more advanced Kaposi’s sarcoma (KS, another KSHV-caused malignancy) lesions containing a greater number of KSHV-infected cells [21]. Moreover, xCT can be upregulated by KSHV-microRNAs by directly targeting BACH-1, one of the negative transcription regulators of xCT, thereby facilitating viral dissemination and persistence in the host [21]. In the same study, we also report that xCT protects KSHV-infected endothelial cells from death induced by reactive oxygen species (ROS) [21]. In another our recent study, we report that xCT is able to activate intracellular signaling pathways especially MAPK, cytokine release and cell invasiveness through induction of 14-3-3β protein [22]. Even though these understandings about xCT and KSHV pathogenesis, it remains unclear whether xCT is also expressed on KSHV-infected PEL tumor cells and its functions in PEL pathogenesis especially tumor cells growth/survival and underlying complex mechanisms. More importantly, it is interested to know whether targeting xCT may represent a promising therapeutic strategy against PEL tumor progression *in vivo*.

## Results

### xCT is highly expressed on KSHV-infected PEL cell-lines

**Figure 1:**
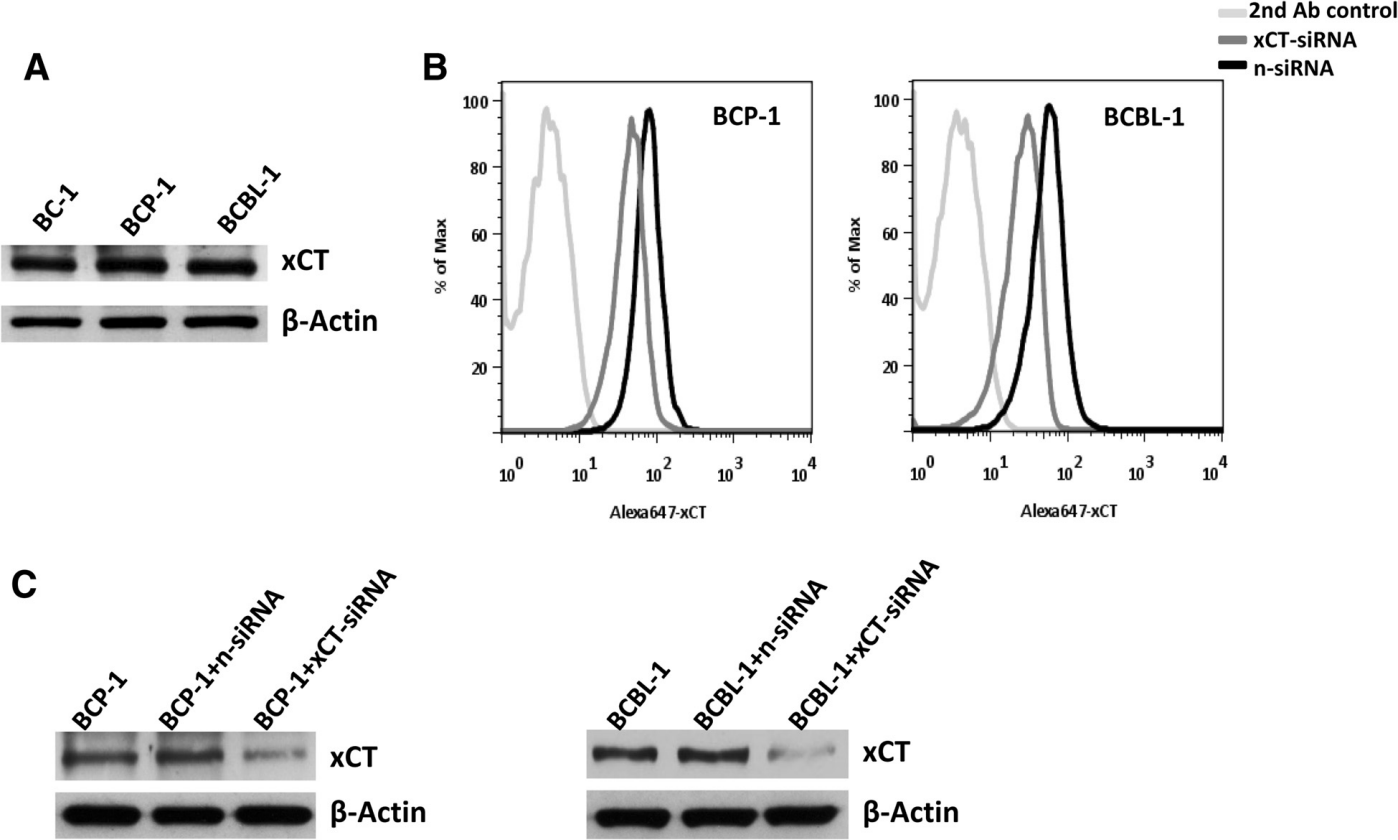
**xCT is highly expressed on KSHV-infected PEL cells. (A)** Immunoblots were performed to identify xCT expression for 3 KSHV-infected PEL cell lines (BC-1, BCP-1 and BCBL-1). Expression of β-actin was assessed for loading controls. **(B-C)** PEL cells were transfected with negative control siRNA (n-siRNA) or siRNA targeting xCT for 48 h. Cells were then incubated with a monoclonal Ab recognizing an extracellular domain of xCT, followed by a secondary Ab conjugated to Alexa-647. PEL cell surface expression of xCT was then quantified by flow cytometry **(B)**. The total xCT expression was measured by immunoblots **(C)**.

To first identify whether xCT is expressed on KSHV-infected PEL cells, we tested its expression in 3 KSHV-infected PEL cell-lines BC-1, BCP-1 and BCBL-1 (BC-1 was also EBV^+^) using immunoblots. Our data demonstrated the expression of xCT in all 3 KSHV-infected PEL cell-lines but no significant difference at its expressional levels among these cell-lines (Figure 1A). Next, using a monoclonal Ab recognizing an extracellular domain of xCT in flow cytometry, we confirmed that xCT is highly expressed on the cell surface of KSHV-infected PEL cell-lines including BCP-1 and BCBL-1. Moreover, RNAi targeting xCT successfully reduced its expression on cell-surface and total cellular levels (Figure 1B-C). To understand its relevance in AIDS-related lymphoma, we also tested the cell-surface expression of xCT on different lymphoma cell-lines. We found that among 4 Burkitt’s lymphoma cell lines, BL-41 and BJAB (both are KSHV^neg^/EBV^neg^) highly expressed xCT, AKATA (KSHV^neg^/EBV^+^) intermediately expressed xCT while RAMOS (KSHV^neg^/EBV^neg^) was lack of xCT expression (Additional file 1: Figure S1). In addition, one diffuse large cell lymphoma (DLCL) cell line CRL2631 (KSHV^neg^/EBV^neg^) also intermediately expressed xCT on cell-surface (Additional file 1: Figure S1).

### Targeting xCT induces KSHV-infected PEL cell death/apoptosis *in vitro*

**Figure 2:**
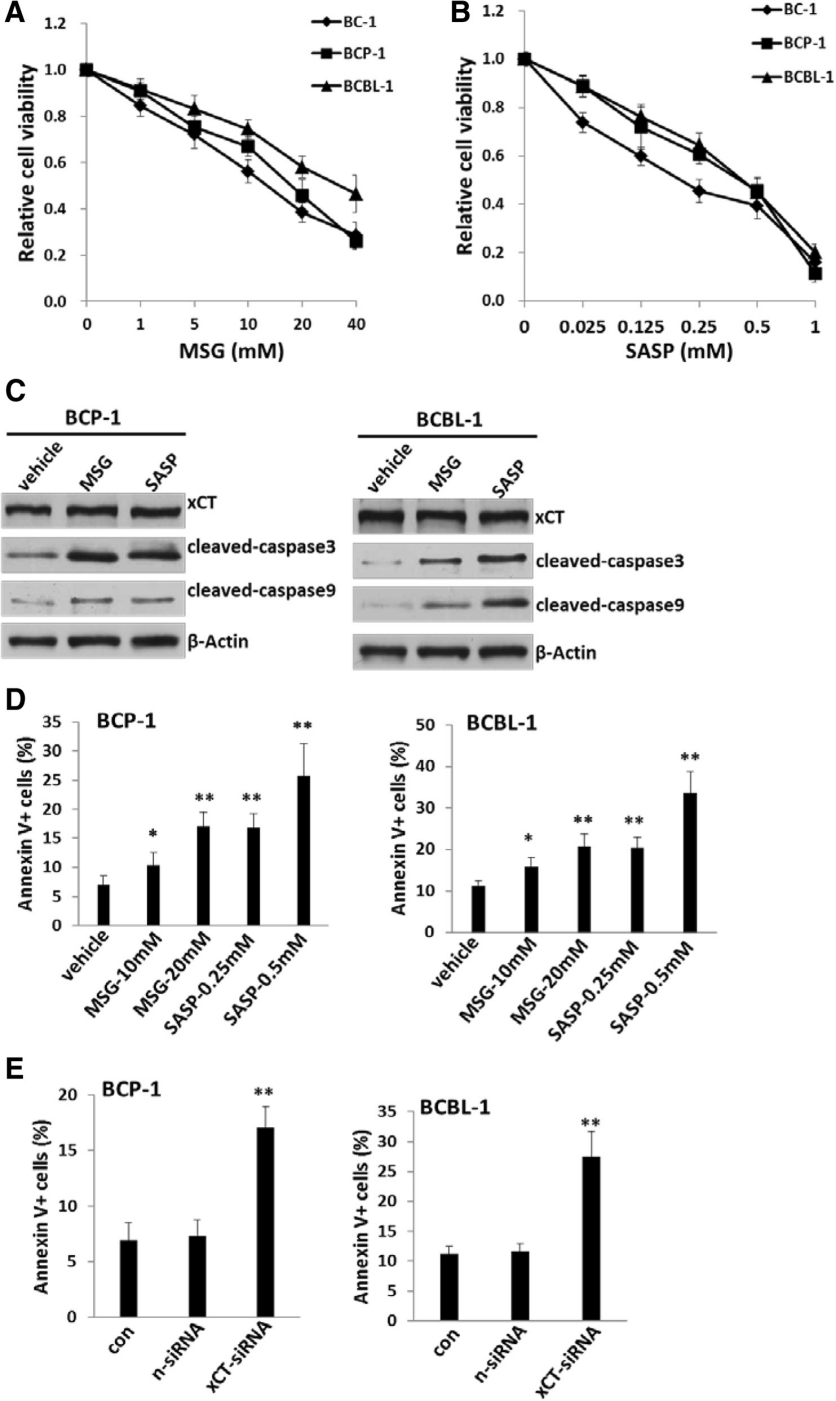
**Targeting xCT induces apoptosis and death for KSHV-infected PEL cells. (A-B)** KSHV-infected BC-1, BCP-1 and BCBL-1 cells were incubated with xCT inhibitor either Monosodium glutamate (MSG), or Sulfasalazine (SASP) at the indicated concentrations for 48 h, then cell viability was assessed by standard MTT assays. **(C)** BCP-1 or BCBL-1 were treated with MSG (20 mM), SASP (0.5 mM) or vehicle for 24 h, then proteins expression were measured by immuoblots. **(D-E)** BCP-1 or BCBL-1 were incubated with MSG or SASP at the indicated concentrations for 24 h **(D)**, or transfected with negative control or xCT-siRNA for 48 h **(E)**. Cell apoptosis was assessed using Annexin V-PI staining and flow cytometry analysis. Error bars represent the S.E.M. for 3 independent experiments. * = p < 0.05; ** = p < 0.01.

To investigate the role of xCT in PEL cell growth/survival, we treated them with two well-known xCT specific inhibitors [13], Monosodium glutamate (MSG) and Sulfasalazine (SASP). After 48-h treatment, both MSG and SASP significantly reduced cell viability for BC-1, BCP-1 and BCBL-1 in a dose-dependent manner by using MTT assays (Figure 2A-B). Further experiments demonstrated that MSG or SASP treatment increased pro-apoptotic cleaved-caspase 3 and 9 expression, while not changing xCT itself expression within BCP-1 and BCBL-1 (Figure 2C). Flow cytometry analysis also indicated that MSG or SASP treatment only slightly reduced xCT expression on the surface of these cells (Additional file 1: Figure S2), implying that both drugs mainly block the functions of xCT but not affecting its expression or cellular location. By using Annexin-PI staining, we confirmed that both MSG and SASP treatment induced BCP-1and BCBL-1 cells undergoing apoptosis in a dose- and time-dependent manner (Figure 2D, Additional file 1: Figure S3). To exclude MSG- or SASP-induced cell apoptosis is due to non-specific “off-target”, we used RNAi assays to directly silence xCT in PEL cells. As shown in Figure 2E, “knock-down” xCT significantly induced cell apoptosis for BCP-1 and BCBL-1, confirming the role of xCT in PEL cell growth/survival. Additional data indicated that inhibition of xCT by SASP also induced significant apoptosis for BL-41 lymphoma cells with highly expressed xCT, while had very slight effects on RAMOS lymphoma cells with deficient xCT expression (Additional file 1: Figure S1 and S4).

### Inhibition of xCT reduces intracellular glutathione (GSH) but increasing reactive oxygen species (ROS) from KSHV-infected PEL cells

**Figure 3:**
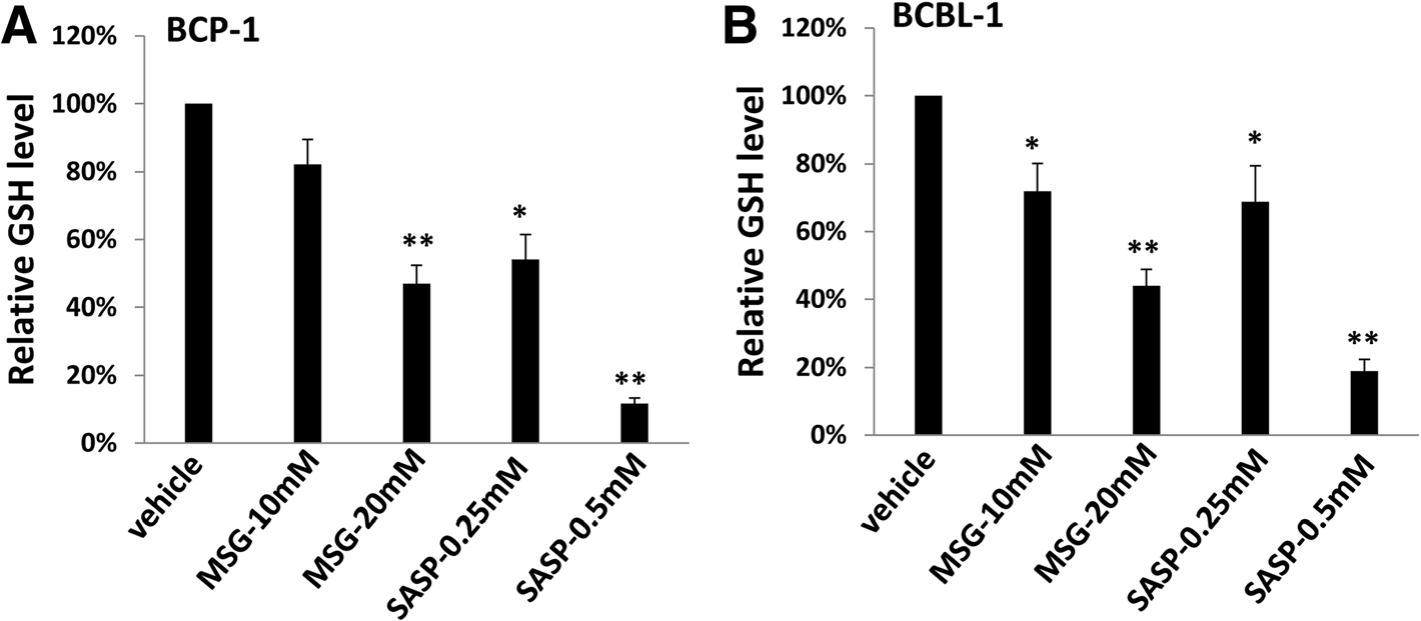
**xCT inhibition reduces intracellular GSH levels from KSHV-infected PEL cells. (A-B)** BCP-1 **(A)** and BCBL-1 **(B)** cells were incubated with either MSG or SASP at the indicated concentrations for 24 h, then intracellular glutathione (GSH) was quantified using a commercial kit as described in Methods, and normalized to GSH levels for the vehicle-incubated cells. Error bars represent the S.E.M. for 3 independent experiments. * = p < 0.05; ** = p < 0.01.

**Figure 4:**
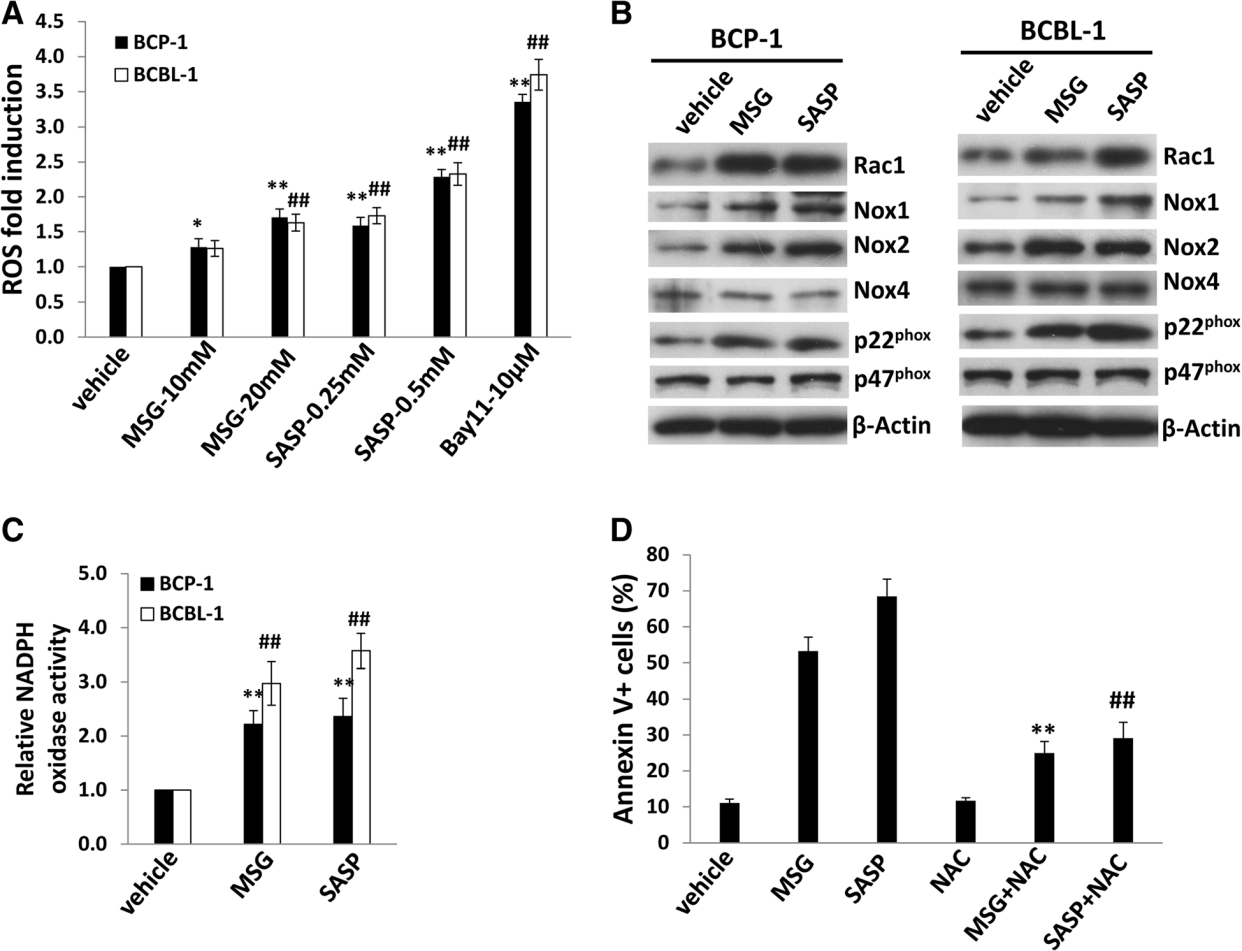
**xCT inhibition induces intracellular ROS levels through upregulation of NADPH oxidases activities from KSHV-infected PEL cells. (A)** BCP-1 and BCBL-1 were incubated with either MSG or SASP at the indicated concentrations for 24 h, then intracellular reactive oxygen species (ROS) were quantified using the ROS-specific dye CM-H2DCFDA and flow cytometry, and normalized to ROS levels for the vehicle-incubated cells. NF-κB inhibitor Bay11-7082 was used as a positive control. **(B-C)** BCP-1 and BCBL-1 were treated with either MSG (20 mM), SASP (0.5 mM) or vehicle for 24 h, then proteins expression were measured by immuoblots. NADPH oxidases activities were measured as described in Methods. **(D)** BCBL-1 were treated with either MSG (20 mM), SASP (0.5 mM) or vehicle in the presence or absence of the antioxidant *N*-acetylcysteine (NAC) 2.5 mM for 48 h, then cell apoptosis was assessed using Annexin V-PI staining and flow cytometry analysis (** *vs* MSG group; ## *vs* SASP group). Error bars represent the S.E.M. for 3 independent experiments. * = p < 0.05; **/## = p < 0.01.

As mentioned above, expression of xCT on the cell membrane is essential for the uptake of cystine required for intracellular GSH synthesis, which plays an important role in maintaining the intracellular redox balance [11,12]. Therefore, we sought to determine whether MSG or SASP treatment affected intracellular GSH and ROS levels for KSHV-infected PEL cells. By using a commercial GSH-assay kit, we found that both drugs apparently reduced intracellular GSH from BCP-1 and BCBL-1 cells, especially SASP (Figure 3). In contrast, impaired GSH synthesis by these xCT inhibitors increased intracellular ROS production within PEL cells (Figure 4A), shown by using a ROS-specific dye, 5-(and-6)-chloromethyl-2′,7′-dichlorodihydrofluorescein diacetate, acetyl ester (CM-H2DCFDA) [23]. Here we used one of NF-κB inhibitors, Bay11-7082 as a positive control, because published data have shown blocking NF-κB pathway increasing ROS production and inducing cell apoptosis from KSHV-infected PEL [23]. As we know, ROS production requires the NADPH oxidase complex, which contains various NADPH oxidases and cytosolic components depending on the stimulus signals and cell types [24,25]. Our further data demonstrated that inhibition of xCT mainly upregulated the expression of Rac1/p22^phox^/Nox1/Nox2 axis for ROS production, although the elevated levels are subtle different between BCP-1 and BCBL-1 cells, while very little impacts on p47^phox^ and Nox4 proteins (Figure 4B). In functional validation, we confirmed that MSG and SASP treatment significantly elevated the activities of NADPH oxidases within BCP-1 and BCBL-1 (Figure 4C), using a luminescence-based biochemical assay as described previously [26]. In parallel, we found that RNAi directly silencing xCT also increased intracellular ROS production from BCBL-1 cells potentially through activating the Rac1/ p22^phox^/Nox1/Nox2 axis (Additional file 1: Figure S5). To block ROS production, we next employed one of H₂O₂ scavengers, the antioxidant *N*-acetylcysteine (NAC). We found that NAC treatment significantly reduced BCBL-1 cell apoptosis induced by xCT inhibitors MSG and SASP (Figure 4D), although which cannot completely prevent the induced cell apoptosis implying that other ROS-independent mechanisms are potentially involved.

### Akt complex activities are reduced by xCT inihibitors

**Figure 5:**
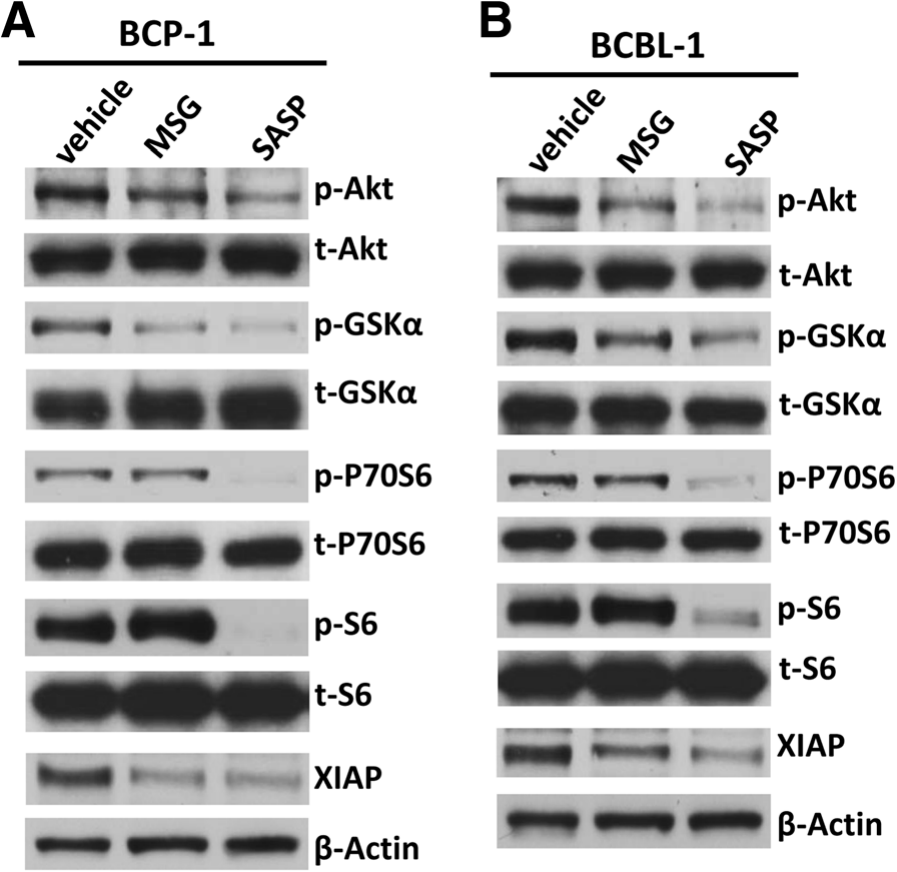
**Targeting xCT blocks the Akt pathway and related protein expression. (A-B)** BCP-1 **(A)** and BCBL-1 **(B)** cells were treated with either MSG (20 mM) or SASP (0.5 mM) for 24 h, then protein expression were measured by immuoblots.

The Akt pathway and related molecules have been found essential for maintaining growth/survival of KSHV-infected PEL cells [7,27]. Blocking Akt complex activities by a variety of compounds induced PEL cell apoptosis and tumor progression *in vitro* and *in vivo*[9,28,29]. Here we found that both MSG and SASP treatment reduced the phosphorylation of Akt and downstream GSKα from BCP-1 and BCBL-1 (Figure 5). Moreover, SASP also effectively repressed the phosphorylation of P70S6 and S6, the two important downstream intermediates of Akt pathway [7]. We also looked at another protein, X-linked inhibitor of apoptosis protein (XIAP), a physiologic substrate of AKT that is stabilized to inhibit programmed cell death and has a direct effect on caspase-3 and 9 [30]. Our data indicated that XIAP expression was greatly reduced in MSG- and SASP-treated PEL cells when compared with vehicle-treated cells (Figure 5). In parallel, RNAi directly silencing xCT also effectively reduced the phosphorylation of Akt from BCBL-1 cells (Additional file 1: Figure S5B). Taken together, these data demonstrate that the Akt pathway is indeed involved in the regulation of PEL growth/survival by xCT.

### Inhibition of xCT induces viral lytic gene expression from KSHV-infected PEL cells

**Figure 6:**
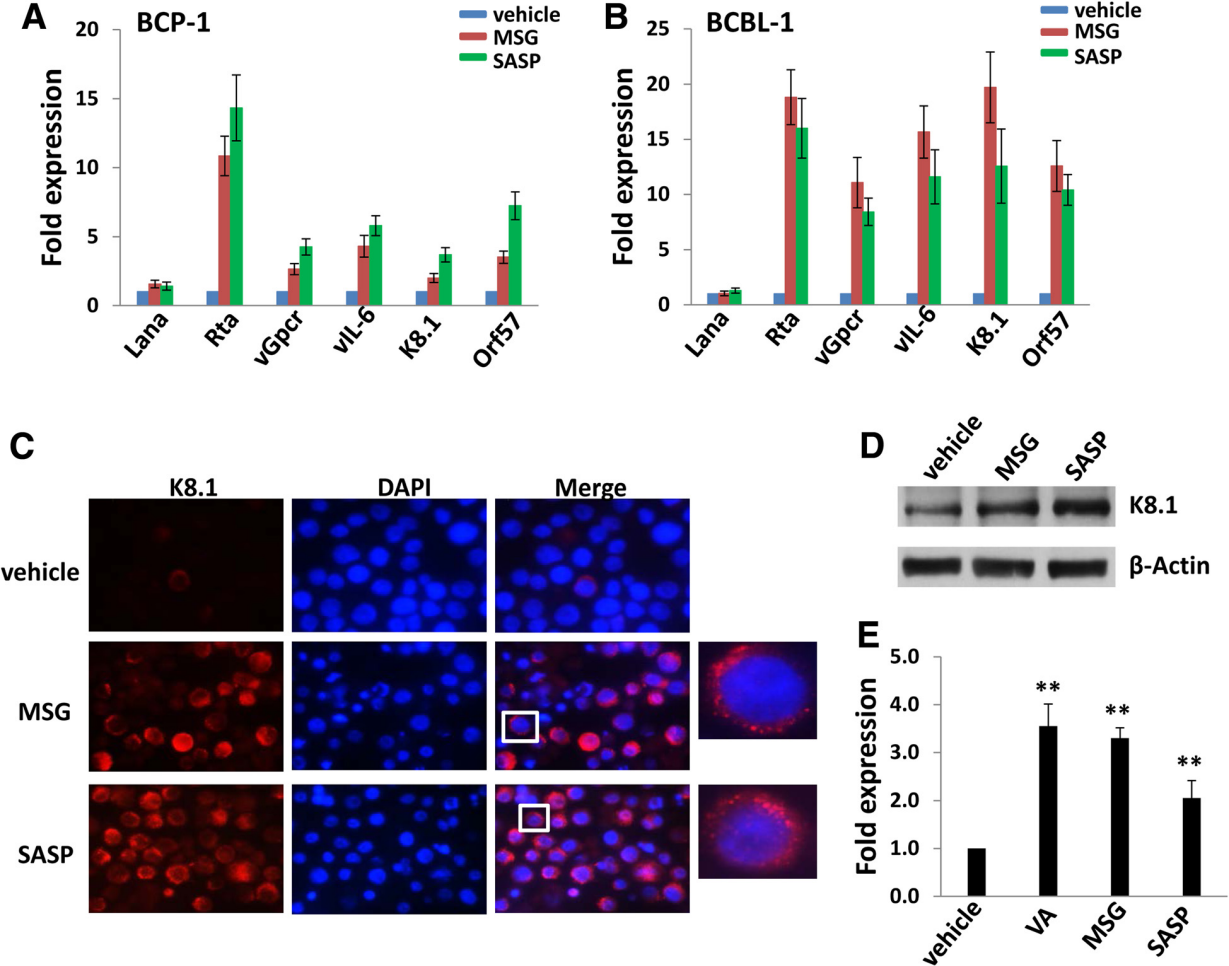
**Inhibition of xCT induces viral lytic gene expression from KSHV-infected PEL cells. (A-B)** BCP-1 **(A)** and BCBL-1 **(B)** cells were treated with either MSG (20 mM), SASP (0.5 mM) or vehicle for 24 h, then viral latent (*Lana*) and lytic gene (*Rta, vGpcr, vIL-6, K8.1, Orf57*) transcripts were quantified using qRT-PCR. Error bars represent the S.E.M for three independent experiments. **(C-D)** BCBL-1 were treated as above, then the expression of viral lytic protein K8.1 was detected by immunofluorescence and immunoblots. **(E)** BCBL-1 were treated by vehicle, MSG (20 mM), SASP (0.5 mM) and valproic acid (1.5 mM as a positive control), respectively, for 48 h, then the virion production were collected as described in Methods, followed by infection of HUVEC cells. *Lana* transcripts from each group were quantified by qRT-PCR. Error bars represent the S.E.M. for 3 independent experiments, ** = p < 0.01.

PEL tumor cells are usually latently infected by KSHV with consistent expression of several viral latent proteins and microRNAs [31]. Therefore, it is interested to know whether inhibition of xCT has impacts on viral gene profile within these cells. Notably, we found that both MSG and SASP treatment dramatically induces viral lytic gene expression, including lytic ‘switch” gene *Rta* and *vGpcr*, *vIL-6*, *K8.1*, *Orf57*, while slightly increased latent gene *Lana* expression within BCP-1 and BCBL-1 (Figure 6A-B). To confirm qRT-PCR results, we detected one of viral lytic ptroteins, K8.1 expression using IFA and immunoblots. As shown in Figure 6C-D, K8.1 expression was significantly increased in the cytoplasma of MSG- or SASP-treated BCBL-1 cells, while only low-level of basal expression was observed in vehicle-treated cells. In parallel, RNAi directly silencing xCT also significantly induced viral lytic gene expression such as *Rta*, *vGpcr* and *K8.1* from BCBL-1 cells (Additional file 1: Figure S6). In addition, we found that inhibition of xCT by MSG and SASP caused virion production from partial BCBL-1 cells (especially MSG) when compared with valproic acid, a well-known KSHV lytic chemical inducer (Figure 6E).

### xCT inhibitor suppresses PEL tumor progression *in vivo*

**Figure 7:**
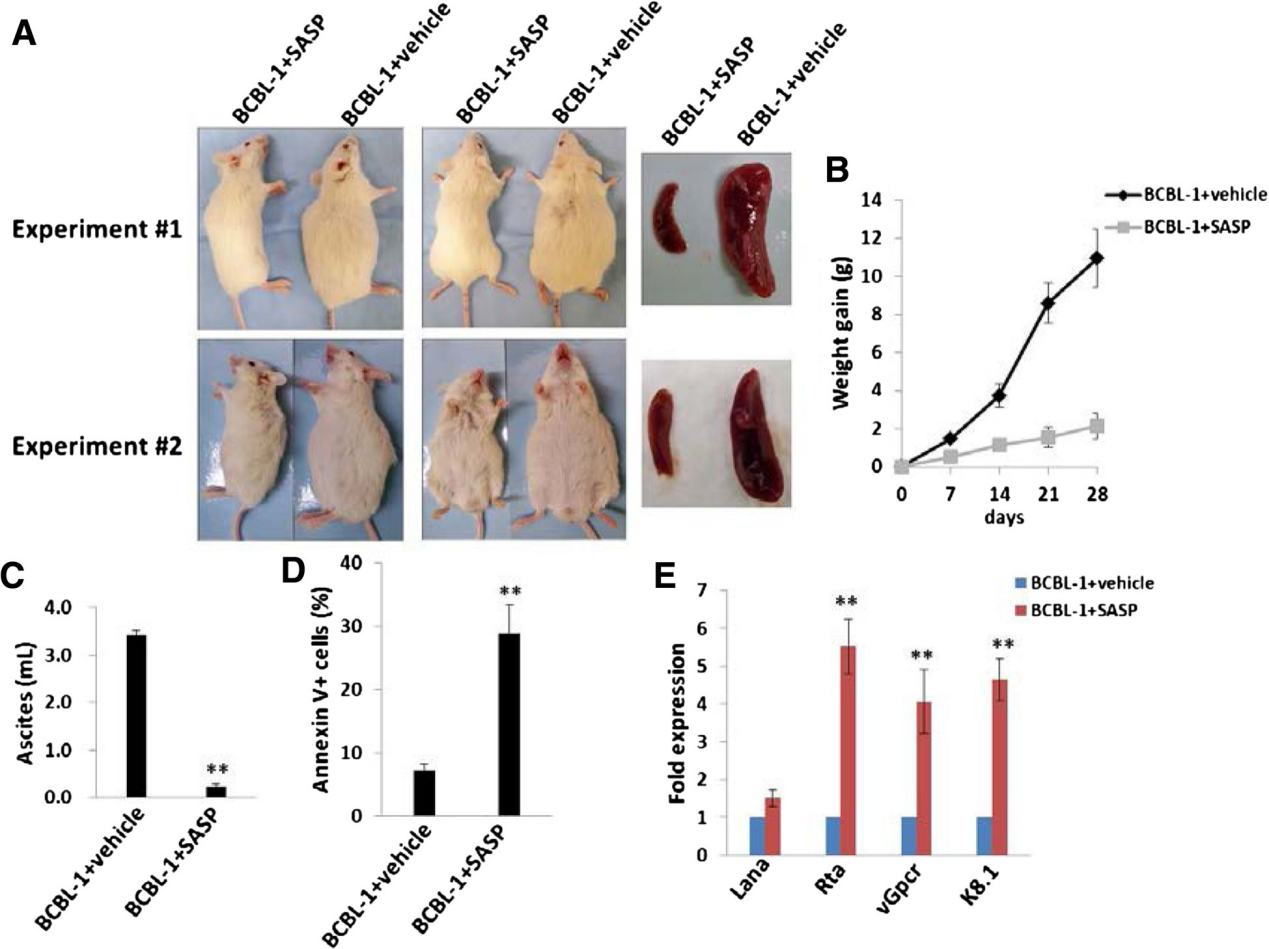
**xCT inhibitor SASP suppresses PEL progression *in vivo*. (A-C)** NOD/SCID mice were injected intraperitoneally (i.p.) with 10^7^ BCBL-1 cells. Beginning 24 h later, 150 mg/kg SASP or vehicle (n = 8 per group) were administered intraperitoneally (i.p.) once daily, 5 days per week, for each of 2 independent experiments. Images of representative animals and their respective spleens, as well as ascites fluid volumes, were collected at the conclusion of experiments on day 28 **(A/C)**. Weights were recorded weekly **(B)**. Error bars represent the S.E.M. for 2 independent experiments, ** = p < 0.01. **(D)** Apoptosis was quantified by flow cytometry for live PEL cells purified from ascites fractions as described in Methods. **(E)** RNA was recovered from live PEL cells from ascites fractions from each of 4 mice representing vehicle- or drug-treated groups. qRT-PCR was used to quantify viral transcripts representing latent (*Lana*) or lytic genes (*Rta, vGpcr, K8.1*). Data were normalized to samples representing vehicle-treated mice.

As noted above, the xCT inhibitors MSG and SASP induced significant cell-apoptosis for KSHV-infected PEL *in vitro*. Unfortunately, MSG is a neurotoxin that precludes its use as a therapeutic agent [32]. In contrast, SASP has been routinely used in the clinic to treat inflammatory bowel disease and rheumatoid arthritis [13], induces cystine starvation and growth arrest in a variety of cancer cells *in vitro* and *in vivo*[13,15,16,33,34]. However, to our knowledge its activity against virus-associated tumors including PEL has not been explored. Therefore, we sought to determine the activity of SASP against PEL tumors *in vivo* utilizing an established xenograft model wherein PEL cells are introduced into the peritoneal cavity of immune compromised mice [9]. In this model, we injected BCBL-1 cells into NOD/SCID mice and observed clear PEL expansion within 3–4 weeks post-injection, including time-dependent weight gain and increased abdominal girth, as well as ascites accumulation and splenic enlargement due to tumor infiltration at the time of necropsy [9]. In the current study, we administered SASP (150 mg/kg) or vehicle i.p. within 24 hours of PEL cell injection. SASP treatment dramatically suppressed PEL tumor progression *in vivo*, including reduced weight/tumor gain, ascites formation and splenic enlargement when compared with the vehicle-treated mice (Figure 7A-C). Using the ascites tumor cells collected and purified from vehicle- or SASP-treated mice, we found that there were much higher apoptotic cell subpopulation in ascites from SASP-treated mice than those from the vehicel-treated group (Figure 7D). We also quantified viral gene profile of ascites-derived PEL cell lysates from each group. We found that PEL cells from SASP-treated mice exhibited significantly increased expression of viral lytic genes profiled (*Rta*, *vGpcr*, *K8.1*), while slightly increasing the latent *Lana* transcripts (Figure 7E).

## Discussion

**Figure 8:**
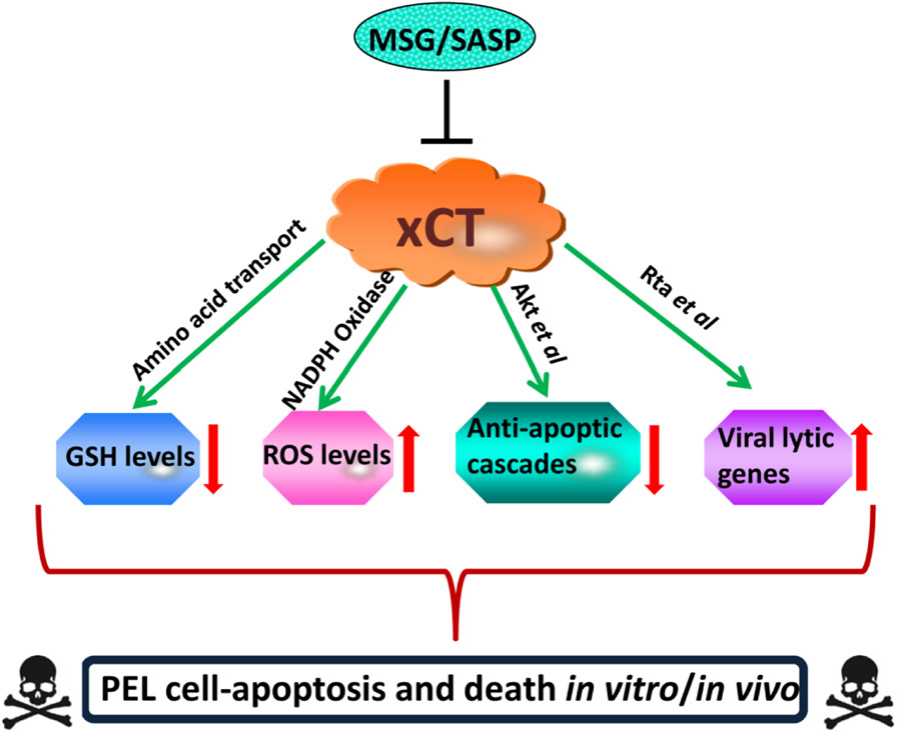
The model of mechanisms for inducing KSHV-infected PEL cell apoptosis and death by targeting xCT.

As mentioned above, our published data demonstrated that xCT is highly expressed in advanced KS tumor tissues from patients and important for KSHV pathogenesis [21,22]. However, in another major KSHV-related malignancy, PEL, it is unclear whether xCT is expressed and functional for tumor progression. In the current study, we provide experimental evidence that xCT is crucial for PEL cell growth/survival and tumor expansion *in vivo* through the complex mechanisms involving both host and viral factors (Figure 8). Our data also indicate that targeting xCT alone or combination of chemotherapy may represent a promising strategy against PEL in future.

xCT expression is differentially regulated during oxidative stress through transcription factors binding to the *cis*-acting “Antioxidant Response Element” (ARE) in its promoter [35,36]. Transcription factors that bind to the ARE include a positive regulator known as Nuclear factor erythroid 2-related factor-2 (Nrf-2) [36] and negative regulators, including BACH-1 and c-Maf, which competitively reduce Nrf-2 binding to the ARE thereby repressing ARE-mediated gene expression [37,38]. We and others have confirmed that at least one KSHV-microRNA, miR-K12-11, can directly target BACH-1 to increase xCT expression [21,39,40]. In addition, c-Maf can also be directly targeted by several KSHV-microRNAs to promote endothelial cell reprogramming [41]. Therefore, it is interested to understand whether these KSHV-microRNAs expressed within PEL cells represent the major contributor to xCT expression and its functions in future studies.

Our data indicate that induction of PEL apoptosis by targeting xCT is potentially through repressing intracellular GSH, increasing ROS and viral lytic gene expression. In accordance with this, Li *et al.* have reported that depletion of GSH and upregulation of ROS strongly induce KSHV reactivation and final cell death for KSHV-infected PEL *in vitro*[23]. The authors also demonstrate that ROS is upregulated by NF-κB inhibition and is required for subsequent KSHV reactivation. However, we do not observe apparent NF-κB inhibition in MSG- or SASP-treated PEL cells when compared with vehicle-treated control (data not shown), implying NF-κB signaling is not responsible for disruption of redox balance by targeting xCT. On the other hand, it has been reported that ROS-induction by KSHV plays a causal role in KS oncogenesis by promoting proliferation and angiogenesis [42]. Furthermore, Rac1 is overexpressed in AIDS-KS lesions and in KSHV-infected mECK36 tumors, and the antioxidant NAC was able to completely suppress Rac1-induced tumor formation in RacCA transgenic mice [43]. Interestingly, published literature indicates a close association between ROS upregulation and Akt signaling activation in a variety of cells [44-46]. In the contrary, we have found here that Akt signaling is greatly impaired while ROS production increased in MSG- or SASP-treated PEL cells. We assume the presence of other compensatory mechanisms for ROS upregulation when Akt signaling is repressed by inhibition of xCT, although which requires further experimental validation.

In fact, xCT is involved in more cellular functions including multidrug resistance for cancer cells. The x_c_^−^ transporter can mediate cellular uptake of cystine to enhance biosynthesis of glutathione, which has a major role in the protection of cells from drug-induced oxidative stress by mediating detoxification of drugs and their extrusion via multidrug resistance proteins [17,18,47-50]. For instance, glutathione induces a conformational change within the multidrug resistance-associated protein-1 (MRP1) and impairs its interaction with a drug and subsequent extrusion function [51]. In a pharmacogenomics approach, Huang *et al.* reported that linking expression of xCT with potency of 1,400 candidate anticancer drugs identified 39 showing positive correlations, and 296 with negative correlations [18]. Interestingly, we recently identify a membrane-protein-complex including Emmprin (CD147), LYVE-1 (a hyaluronan receptor) and BCRP (a drug-efflux pump protein), responsible for multidrug resistance of KSHV-infected PEL cells [52,53]. Therefore, future work will focus on determining whether xCT is also involved in this protein-complex to mediate multidrug resistance for PEL. Finally, it is interested to identify more cellular genes within PEL cells potentially regulated by xCT, through analysis of the global gene profile changed due to inhibition of xCT using “-omics” technologies.

## Materials and methods

### Cell culture and reagents

Body cavity-based lymphoma cells (BCBL-1, KSHV^+^/EBV^neg^) and a Burkitt’s lymphoma cell line BL-41 (KSHV^neg^/EBV^neg^) were kindly provided by Dr. Dean Kedes (University of Virginia) and maintained in RPMI 1640 medium (Gibco) with supplements as described previously [9]. The Burkitt’s lymphoma cell line BJAB (KSHV^neg^/EBV^neg^), RAMOS (KSHV^neg^/EBV^neg^), AKATA (KSHV^neg^/EBV^+^) were kindly provided by Dr. Erik Flemington (Tulane University) and cultured as described elsewhere [54]. PEL cell line BC-1 (KSHV^+^/EBV^+^), BC-3 (KSHV^+^/EBV^neg^), and BCP-1 (KSHV^+^/EBV^neg^) cells were purchased from American Type Culture Collection (ATCC) and maintained in complete RPMI 1640 medium (ATCC) supplemented with 20% FBS. A diffuse large cell lymphoma (DLCL) cell line CRL2631 (KSHV^neg^/EBV^neg^) was purchased from ATCC and maintained in complete RPMI 1640 medium (ATCC) supplemented with 10% FBS. Primary human umbilical vein endothelial cells (HUVEC) were cultured as described previously [21]. KSHV infection was verified for all cell lines using immunofluorescence assays for detection of the KSHV latency-associated nuclear antigen (LANA) [9]. All cells were cultured at 37°C in 5% CO_2_. All experiments were carried out using cells harvested at low (<20) passages. Monosodium glutamate (MSG), Sulfasalazine (SASP) and Bay11-7082 were purchased from Sigma.

### Cell viability assays

Cell viability was assessed using MTT assays for assessment of proliferative capacity, and flow cytometry for quantitative assessment of apoptosis. The standard MTT assays were performed as described previously [9]. For flow cytometry, the FITC-Annexin V/propidium iodide (PI) Apoptosis Detection Kit I (BD Pharmingen) were used according to the manufacturer’s instructions.

### Immunoblotting

Cells were lysed in buffer containing 20 mM Tris (pH 7.5), 150 mM NaCl, 1% NP40, 1 mM EDTA, 5 mM NaF and 5 mM Na_3_VO_4_. Total cell lysates (30 μg) were resolved by 10% SDS–PAGE, transferred to nitrocellulose membranes, and immunoblotted using 100–200 μg/mL antibodies, including cleaved-caspase 3/9, p-Akt/t-Akt, p-GSKα/t-GSKα, p-P70S6/t-P70S6, p-S6/t-S6, XIAP, Rac1 (cell signaling), Nox1 (Abcam), xCT, p22^phox^, p47^phox^, Nox2, Nox4 (Santa Cruz), KSHV-K8.1 (ABI). For loading controls, blots were reacted with antibodies detecting β-Actin (Sigma). Immunoreactive bands were developed using an enhanced chemiluminescence reaction (Perkin-Elmer) and visualized by autoradiography.

### Immunofluorescence Assays (IFA)

Cells were incubated in 1:1 methanol-acetone at −20°C for fixation and permeabilization, then with a blocking reagent (10% normal goat serum, 3% bovine serum albumin, and 1% glycine) for an additional 30 minutes. Cells were then incubated for 1 h at 25°C with 1:2000 dilution of a mouse anti-K8.1 monoclonal antibody (ABI) followed by 1:200 dilution of a goat anti-mouse secondary antibody conjugated to Texas Red (Invitrogen). For identification of nuclei, cells were subsequently counterstained with 0.5 μg/mL 4′,6-diamidino-2-phenylindole (DAPI; Sigma) in 180 mM Tris–HCl (pH 7.5). Cells were washed once in 180 mM Tris–HCl for 15 minutes and prepared for visualization using a Leica TCPS SP5 AOBS confocal microscope.

### RNA interference

For RNA interference assays, xCT ON-TARGET plus SMART pool siRNA (Dharmacon), or negative control siRNA, were delivered using the DharmaFECT transfection reagent according to the manufacturer’s instructions. To confirm initial transfection efficiency for siRNA experiments, PEL cells were transfected with green fluorescent protein (GFP)-tagged siRNA, and GFP expression determined by flow cytometry 24 h later as described previously [9]. Three independent transfections were performed for each experiment, and all samples were analyzed in triplicate for each transfection.

### qRT-PCR

Total RNA was isolated using the RNeasy Mini kit according to the manufacturer’s instructions (QIAGEN). cDNA was synthesized from equivalent total RNA using SuperScript III First-Strand Synthesis SuperMix Kit (Invitrogen) according to the manufacturer’s procedures. Primers used for amplification of target genes are displayed in Additional file 2: Table S1. Amplification was carried out using an iCycler IQ Real-Time PCR Detection System, and cycle threshold (Ct) values were tabulated in duplicate for each gene of interest in each experiment. “No template” (water) controls were used to ensure minimal background contamination. Using mean Ct values tabulated for each gene, and paired Ct values for β-actin as an internal control, fold changes for experimental groups relative to assigned controls were calculated using automated iQ5 2.0 software (Bio-rad).

### Measurement of virion production

BCBL-1 cells (10 mL) were treated by vehicle, MSG (20 mM), SASP (0.5 mM) and valproic acid (1.5 mM as a positive control), respectively, for 48 h, then followed by centrifugation at 1200 rpm for 5 min and 3000 rpm for 20 min. The clear supernatants were collected followed by ultracentrifugation at 30000 *g* for 2 h, and virion pellet were resuspended at 100 μL of fresh medium. The HUVEC cells were infected with these virion suspensions as described previously [22] and followed by qRT-PCR measurement of *Lana* transcripts mentioned above.

### ROS measurement

Vehicle-, MSG- and SASP-treated PEL cells were loaded with 10 μM of the ROS dye c-H2DCFDA (Invitrogen) for 30 min at 37°C in Hanks’ Balanced Salt Solution (HBSS) containing calcium and magnesium (HBSS/Ca/Mg). Cells will be washed once with HBSS/Ca/Mg to remove dye, resuspended in HBSS/Ca/Mg and subjected to flow cytometry analyses as described elsewhere [23]. To block ROS production, PEL cells were treated with MSG, SASP or vehicle in the presence or absence of the antioxidant *N*-acetylcysteine (NAC) 2.5 mM for 48 h, then cell apoptosis was assessed as described above.

### Flow cytometry

Cells were resuspended in staining buffer (3 BSA in 1× PBS) for 20 minutes, then incubated on ice for 30 min with 1:20 dilution of primary antibody xCT (Santa Cruz). Following two subsequent wash steps, cells were incubated for an additional 30 min with 1:200 dilution of secondary antibodies (Invitrogen) including Donkey anti-goat IgG Alexa Fluor 647. Controls included cells incubated with the secondary antibody only. Cells were resuspended in 1× PBS and analyzed using a FACS Calibur 4-color flow cytometer (BD) and FlowJo software (TreeStar) to quantify cell surface localization of target proteins.

### NADPH oxidase activities assays

The chemiluminescence-based NADPH oxidase activities assays were performed as described previously with modifications [26]. After drug-treatment, cells were centrifuged at 500 g for 10 min at 4°C. The cell pellet was resuspended with 35 μL ice-cold lysis buffer and kept on ice for 20 min. To a final 200 μl of HBSS/Ca/Mg buffer containing NADPH (1 μM, Sigma) and lucigenin (20 μM, Sigma), 5 μl of cell lysates was added to initiate the reaction for 5 min at 37°C. Chemiluminescence was measured immediately using a Synergy HT microplate reader (BioTek Instruments).

### Intracellular GSH measurement

The intracellular GSH levels in vehicle-, MSG- and SASP-treated PEL cells were quantified using the GSH-Glo™ Glutathione Assay Kit (Promega), according to the manufacturer’s instructions.

### PEL xenograft model

BCBL-1 cells maintained at early passage number in cell culture were washed twice in sterile-filtered PBS prior to performance of trypan blue and flow cytometry assays for verification of their viability. Aliquots of 10^7^ viable cells were diluted in 200 μL sterile PBS, and 6–8 week-old male non-obese diabetic/severe-combined immunodeficient (NOD/SCID) mice (Jackson Laboratory, Taconic Inc.) received intraperitoneal (i.p.) injections with a single cell aliquot. SASP solutions were prepared at 20 mg/mL, dissolved in 0.1 N NaOH in PBS at pH 7.2, and sterile-filtered prior to *in vivo* administration. The SASP (150 mg/kg body weight), or vehicle alone, was administered using an insulin syringe for i.p. injection. Drug was administered 24 h after BCBL-1 injection, once daily for 5 days/week. Two experiments, with 8 mice per group for each experiment, were performed. The PEL expansion *in vivo* was confirmed by testing the expression of cell-surface markers including CD45, CD138, EMA and viral protein LANA in nuclear within ascites tumor cells, using IFA and flow cytometry as described in our previous publications [9]. Weights were recorded weekly as a surrogate measure of tumor progression, and ascites fluid volumes were tabulated for individual mice at the completion of each experiment. All protocols were approved by the Louisiana State University Health Science Center Animal Care and Use Committee in accordance with national guidelines.

### Statistical analyses

Significance for differences between experimental and control groups was determined using the two-tailed Student’s t-test (Excel 8.0).

## Competing interests

The authors declare that they have no competing interests.

## Authors’ contributions

Participated in research design: LD, ZQ. Conducted experiments: LD, YC, ZQ. Performed data analysis: LD, YC, ZQ. Wrote or contributed to the writing of the manuscript: LD, YC, CP, ZQ. All authors read and approved the final manuscript.

## Supplementary Material

Additional file 1: Figure S1 xCT expression on cell-surface of AIDS-related lymphoma. **Figure S2.** xCT expression on the surface of vehicle-, MSG- or SASP-treated PEL cells. **Figure S3.** Targeting xCT induces apoptosis for KSHV-infected PEL cells. **Figure S4.** Targeting xCT induces apoptosis for AIDS-related lymphoma. **Figure S5.** Targeting xCT by RNAi induces intracellular ROS levels through upregulation of NADPH oxidases from KSHV-infected PEL cells. **Figure S6.** Targeting xCT by RNAi induces viral lytic gene expression from KSHV-infected PEL cells. Click here for file

Additional file 2: Table S1 Primer sequences for qRT-PCR in this study. Click here for file
