# Supplementary material for: Targeting xCT, a cystine-glutamate transporter induces apoptosis and tumor regression for KSHV/HIV-associated lymphoma
Source: J Hematol Oncol. 2014 Apr 4;7:30. doi: 10.1186/1756-8722-7-30 (PMC4234972; doi:10.1186/1756-8722-7-30)
Supplement: Additional file 1: Figure S1 — xCT expression on cell-surface of AIDS-related lymphoma. Figure S2. xCT expression on the surface of vehicle-, MSG- or SASP-treated PEL cells. Figure S3. Targeting xCT induces apoptosis for KSHV-infected PEL cells. Figure S4. Targeting xCT induces apoptosis for AIDS-related lymphoma. Figure S5. Targeting xCT by RNAi induces intracellular ROS levels through upregulation of NADPH oxidases from KSHV-infected PEL cells. Figure S6. Targeting xCT by RNAi induces viral lytic gene expression from KSHV-infected PEL cells. [file 1756-8722-7-30-S1.docx]

**Figure S1**

**
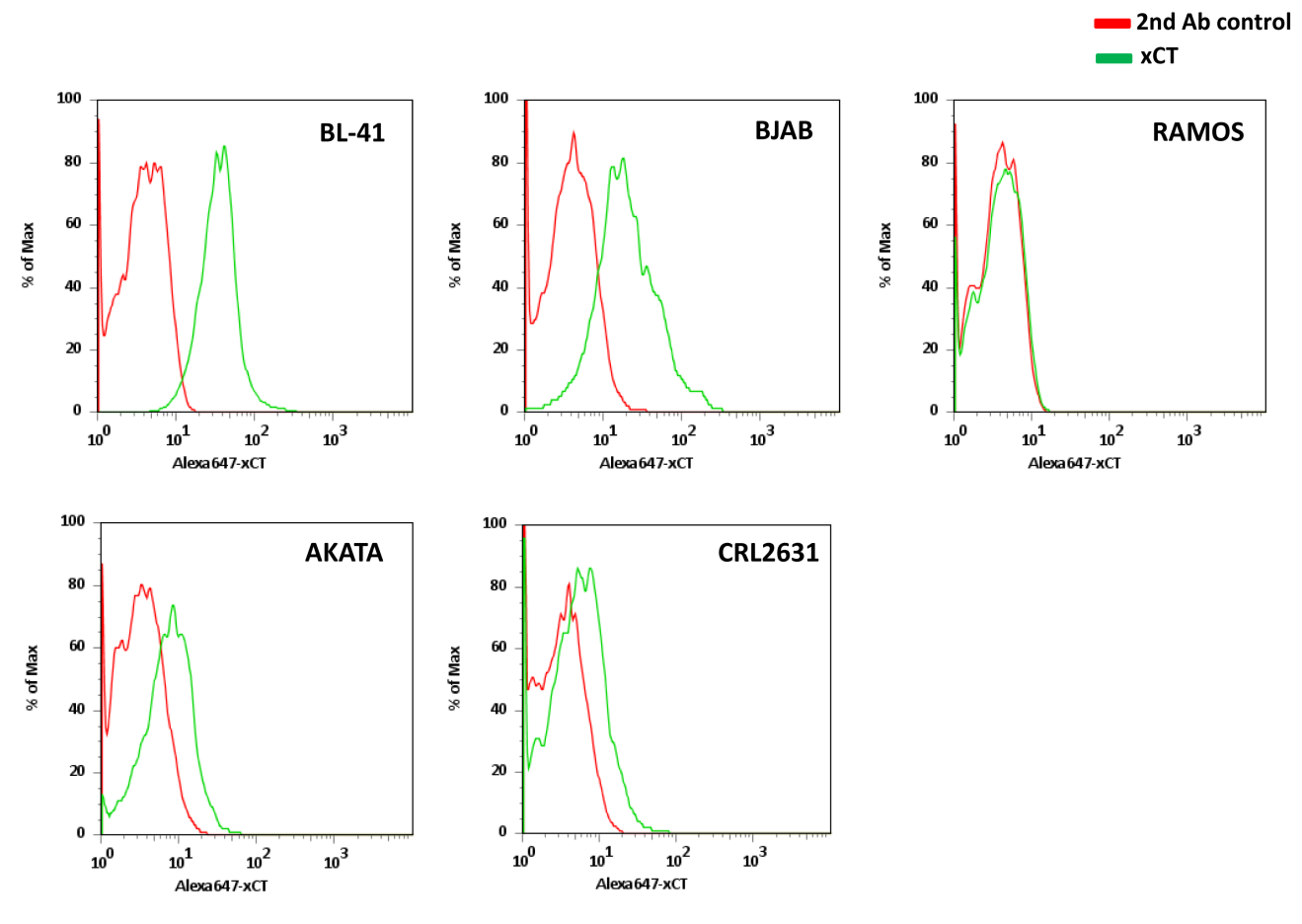
**

**Figure S1. xCT expression on cell-surface of AIDS-related lymphoma.** 4 Burkitt's lymphoma cell lines (BL-41, BJAB, RAMOS, AKATA) and 1 diffuse large cell lymphoma (DLCL) cell line CRL2631 were incubated with a monoclonal Ab recognizing an extracellular domain of xCT, followed by a secondary Ab conjugated to Alexa-647, then cell surface expression of xCT was quantified by flow cytometry.

**Figure S2**


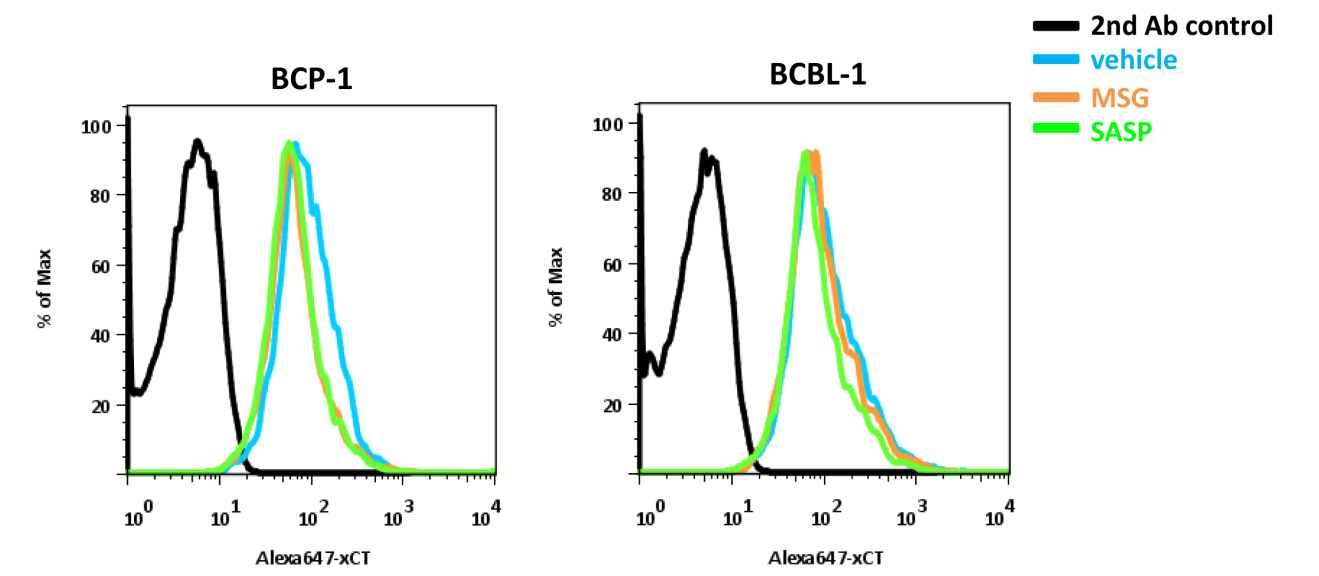


**Figure S2. xCT expression on the surface of vehicle-, MSG- or SASP-treated PEL cells.**  BCP-1 and BCBL-1 were treated with MSG (20 mM), SASP (0.5 mM) or vehicle for 24 h, respectively, then incubated with a monoclonal Ab recognizing an extracellular domain of xCT, followed by a secondary Ab conjugated to Alexa-647. xCT expressed on cell surface was quantified by flow cytometry.

**Figure S3**


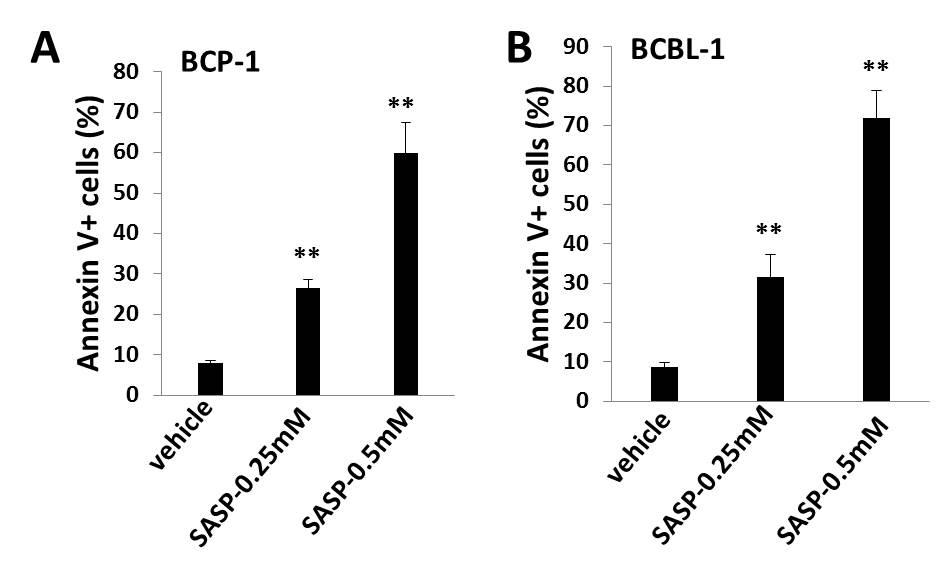


**Figure S3. Targeting xCT induces apoptosis for KSHV-infected PEL cells.** (**A-B**) BCP-1 (A) or BCBL-1 (B) were treated with SASP (0.25 or 0.5 mM) or vehicle for 48 h, then cell apoptosis was assessed using Annexin V-PI staining and flow cytometry analysis. Error bars represent the S.E.M. for 3 independent experiments. ** = p<0.01.

**Figure S4**


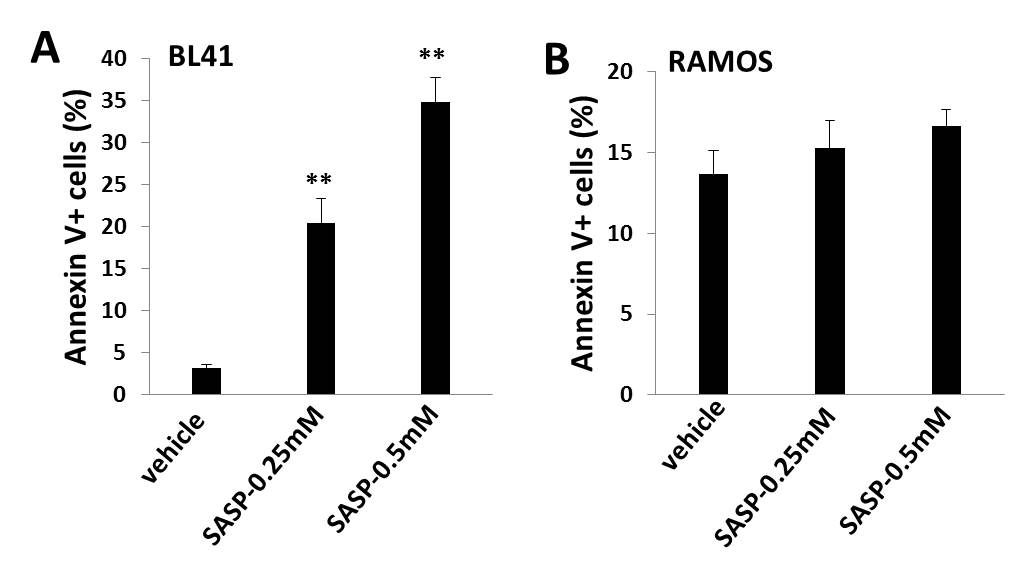


**Figure S4. Targeting xCT induces apoptosis for AIDS-related lymphoma.** (**A-B**) Burkitt's lymphoma cell lines, BL-41 (A) or RAMOS (B) were treated with SASP (0.25 or 0.5 mM) or vehicle for 24 h, then cell apoptosis was assessed using Annexin V-PI staining and flow cytometry analysis. Error bars represent the S.E.M. for 3 independent experiments. ** = p<0.01.

**Figure S5**

**
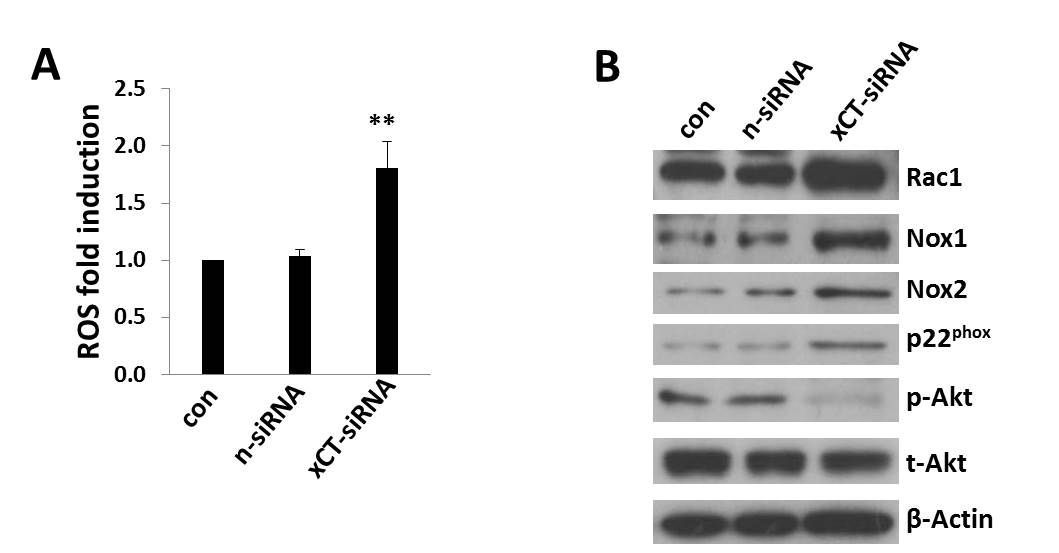
**

**Figure S5. Targeting xCT by RNAi induces intracellular ROS levels through upregulation of NADPH oxidases from KSHV-infected PEL cells.** (**A**) BCBL-1 were transfected with negative control siRNA (n-siRNA) or siRNA targeting xCT for 48h, then intracellular reactive oxygen species (ROS) were quantified using the ROS-specific dye CM-H2DCFDA and flow cytometry, and normalized to ROS levels for the control cells. Error bars represent the S.E.M. for 3 independent experiments. ** = p<0.01. (**B**) Cells were treated as (A), then protein expression were measured by immuoblots.

**Figure S6**

**
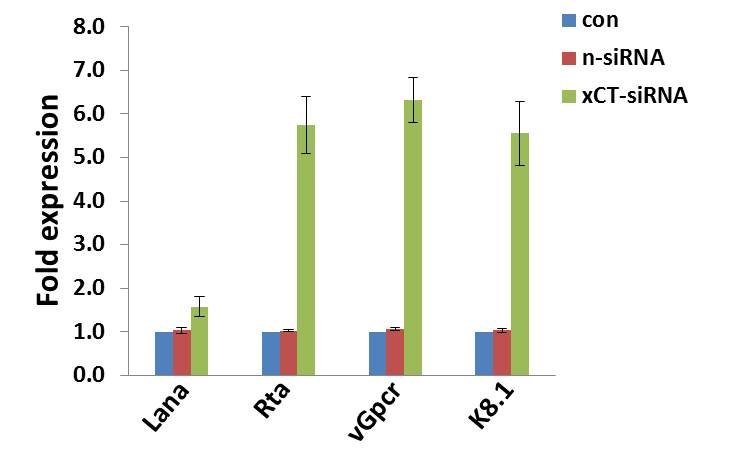
**

**Figure S6. Targeting xCT by RNAi induces viral lytic gene expression from KSHV-infected PEL cells.** BCBL-1 were transfected with negative control siRNA (n-siRNA) or siRNA targeting xCT for 48h, then viral latent (*Lana*) and lytic gene (*Rta, vGpcr, K8.1*) transcripts were quantified using qRT-PCR. Error bars represent the S.E.M for 3 independent experiments.
