# Supplementary material for: Targeting xCT, a cystine-glutamate transporter induces apoptosis and tumor regression for KSHV/HIV-associated lymphoma
Source: J Hematol Oncol. 2014 Apr 4;7:30. doi: 10.1186/1756-8722-7-30 (PMC4234972; doi:10.1186/1756-8722-7-30)
Supplement: Additional file 2: Table S1 — Primer sequences for qRT-PCR in this study. [file 1756-8722-7-30-S2.docx]

**Table S1. Primer sequences for qRT-PCR in this study.**

| **Gene** | **Sequences (5’ 3’)** |
| --- | --- |
| *Lana* | *sense TCCCTCTACACTAAACCCAATA*  *antisense TTGCTAATCTCGTTGTCCC* |
| *Rta* | *sense TAATGTCAGCGTCCACTCC*  *antisense TTCTGGCACGGTCAAAGC* |
| *vIL-6* | *sense ATGTGCTGGTTCAAGTTGTGG*  *antisense GATGGCTGGTAGTTTCAGATG* |
| *vGpcr* | *sense CATCCGCTGCACTGTTAA*  *antisense GCTTTGTCCTCCTCACCA* |
| *K8.1* | *sense CACCACAGAACTGACCGATG*  *antisense TGGCACACGGTTACTAGCAC* |
| *ORF57* | *sense GGGTGGTTTGATGAGAAGGA*  *antisense CGCTACCAAATATGCCACCT* |
| *β-actin* | *sense GGAAATCGTGCGTGACATT*  *antisense GACTCGTCATACTCCTGCTTG* |
